# Supplementary figures and images for: Coordination between terminal variation of the viral genome and insect microRNAs regulates rice stripe virus replication in insect vectors
Source: PLoS Pathog. 2021 Mar 10;17(3):e1009424. doi: 10.1371/journal.ppat.1009424 (PMC7984632; doi:10.1371/journal.ppat.1009424)

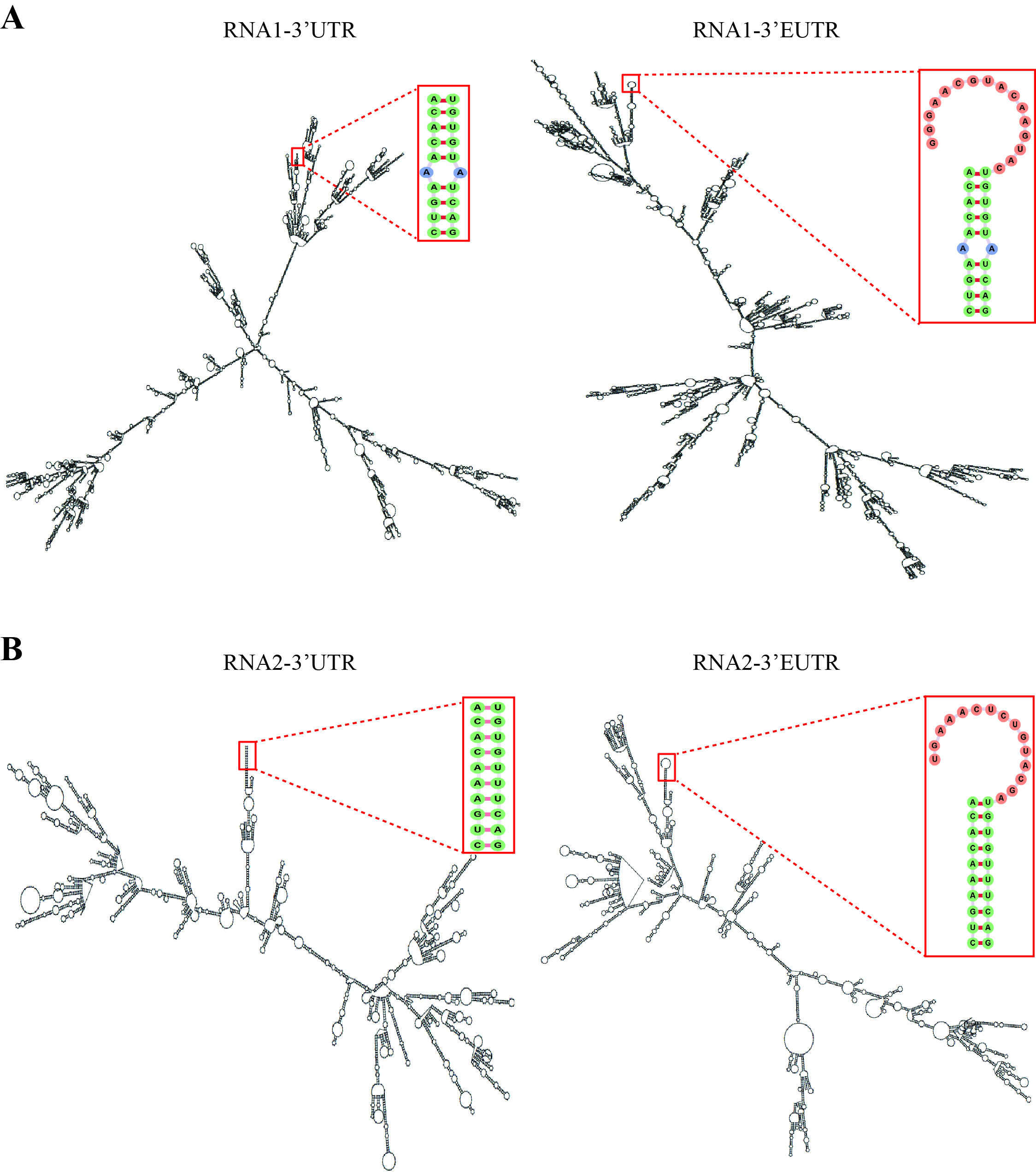

Supplement: S1 Fig — The secondary structure of RSV genomic RNA1 (A) and RNA2 (B) with the regular 3’ untranslated terminal region (3’-UTR) or extended untranslated terminal region (3’-EUTR). The complementary 3’ and 5’ termini are marked with red boxes and enlarged on the right side. (TIF) [file ppat.1009424.s001.tif]

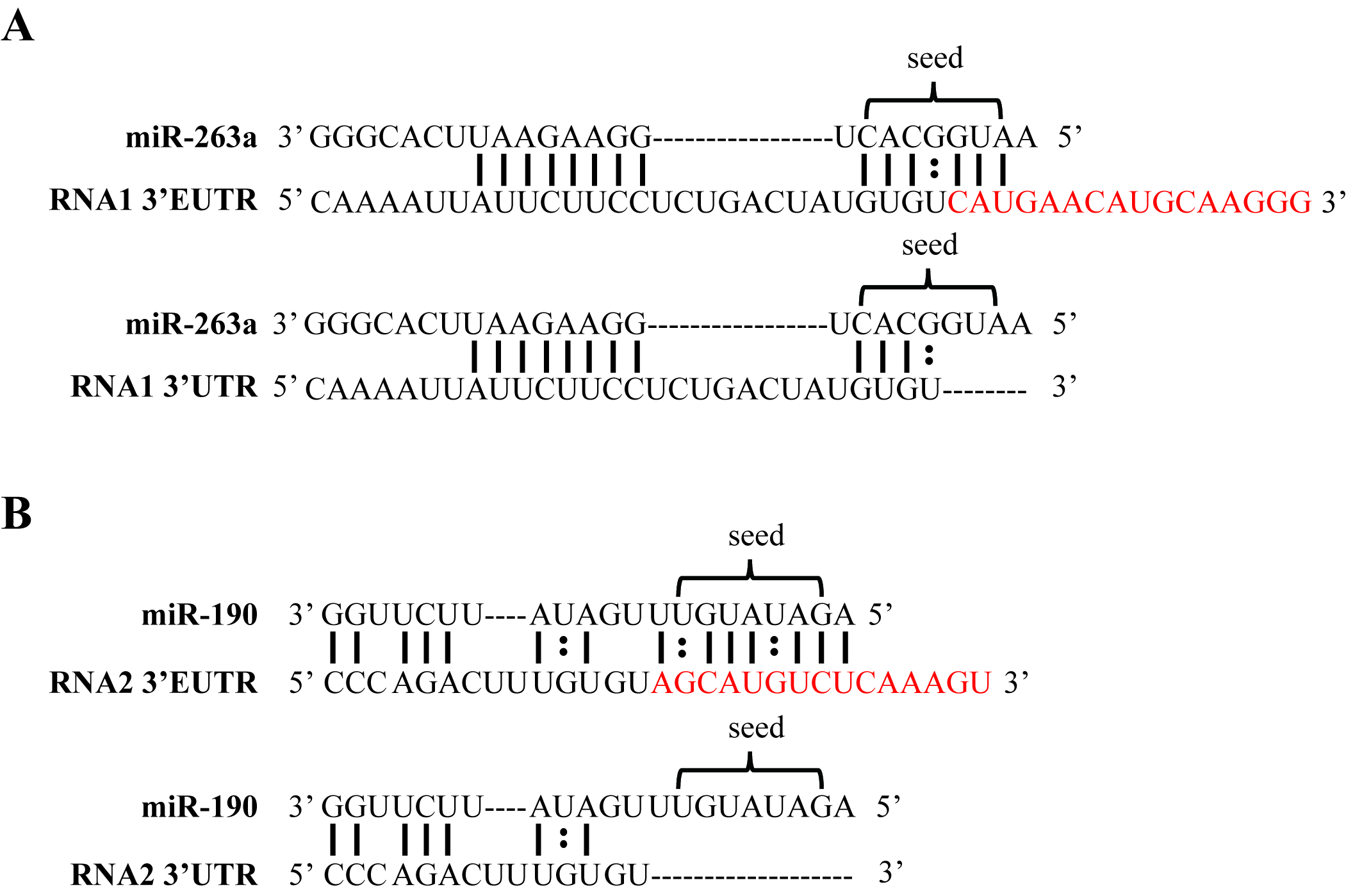

Supplement: S2 Fig — Sequence alignments of miR-263a (A) and miR-190 (B) with the predicted target site in the 3’ terminus of RSV RNA1 or RNA2. The 3’ extensions are shown in red. The G:U wobble pairs are labeled. (TIF) [file ppat.1009424.s002.tif]

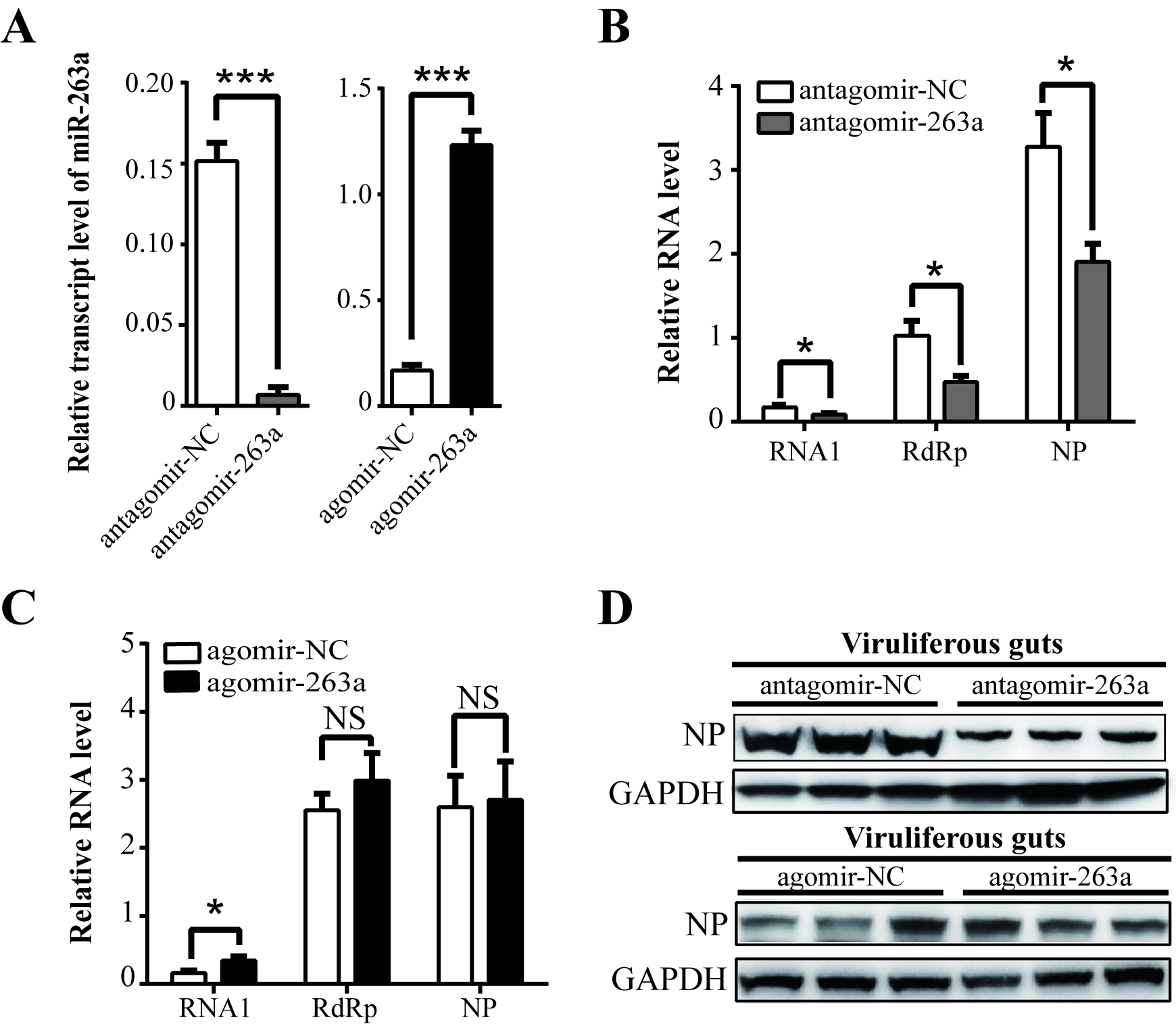

Supplement: S3 Fig — (A) Relative transcript levels of miR-263a in the gut of viruliferous planthopper determined by real-time quantitative PCR (qPCR) 4 d after the injection of antagomir-263a or agomir-263a. Antagomir control (antagomir-NC) or agomir control (agomir-NC) were injected and used as the negative controls. (B) Relative RNA levels of RSV RNA1, RdRp, and NP in the gut of viruliferous planthopper measured by qPCR 4 d after the injection of antagomir-263a or antagomir-NC. (C) Relative RNA levels of RSV RNA1, RdRp, and NP in the gut of viruliferous planthopper measured by qPCR 4 d after the injection of agomir-263a or agomir-NC. The values in (A), (B), and (C) represent the mean ± SE. NS, no significant differences. *, P < 0.05. ***, P < 0.001. (D) Western blot analysis of RSV NP protein in the samples of (B) and (C) using an in-house generated anti-NP monoclonal antibody. GADPH was detected using anti-GADPH polyclonal antibodies and was used as an internal control. (TIF) [file ppat.1009424.s003.tif]

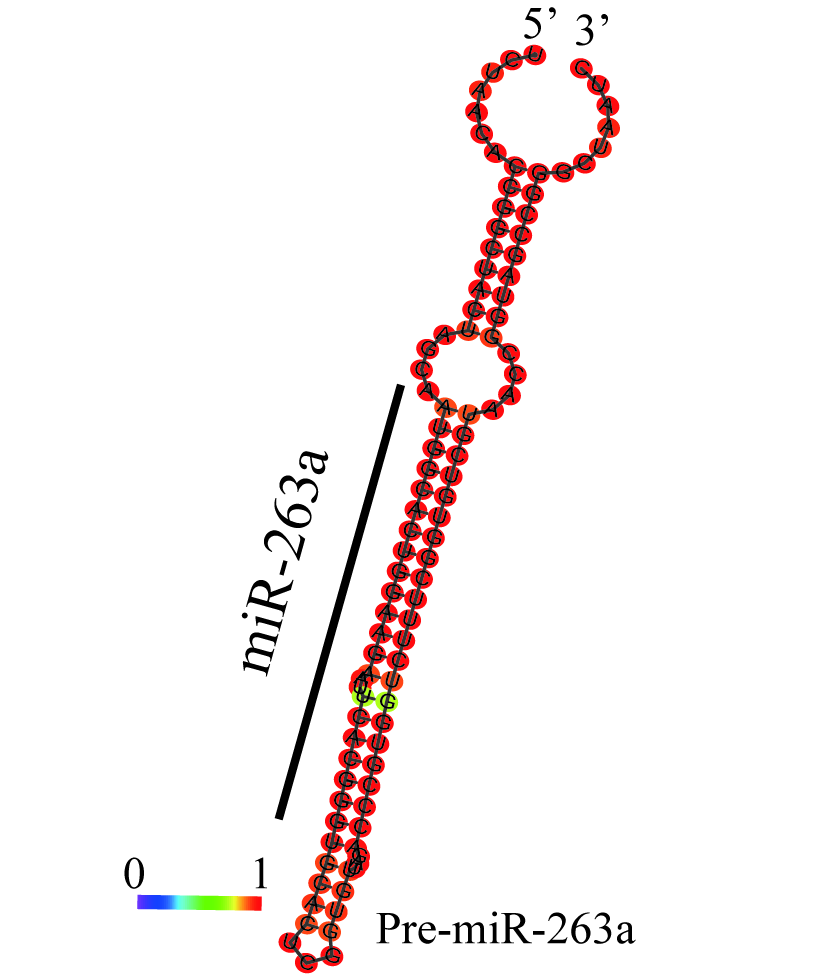

Supplement: S4 Fig — Mature miR-263a is marked with a line. Base-pair probabilities (from 0 to 1) are shown in the heatmap. (TIF) [file ppat.1009424.s004.tif]

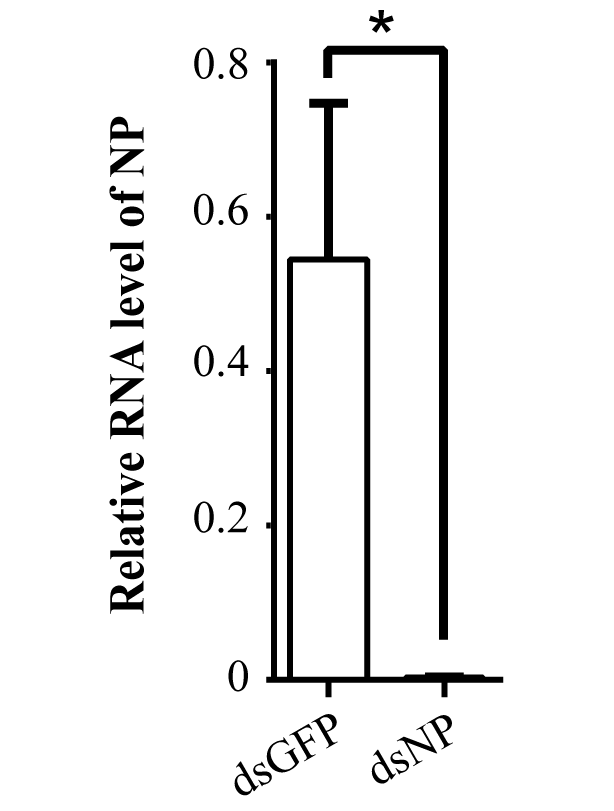

Supplement: S5 Fig — The values were determined by real-time quantitative PCR and are presented as the mean ± SE. *, P < 0.05. (TIF) [file ppat.1009424.s005.tif]
