# Supplementary material for: Coordination between terminal variation of the viral genome and insect microRNAs regulates rice stripe virus replication in insect vectors
Source: PLoS Pathog. 2021 Mar 10;17(3):e1009424. doi: 10.1371/journal.ppat.1009424 (PMC7984632; doi:10.1371/journal.ppat.1009424)
Supplement: S1 Table — (DOCX) [file ppat.1009424.s006.docx]

S1 Table. Primers used in this study.

| Primer name | Sequence (5’ to 3’) |
| --- | --- |
| primers used for qPCR | |
| U6-F | TGGAACGATACAGAGAAGATTAGCA |
| U6-R | AACGCTTCACGATTTTGCGT |
| miR263-q-F | GGCGGGAATGGCACTGGAAGAAT |
| pre-miR263-q-F | CCCGTGGTCTTTCGGTGTCGTAAC |
| pri-miR263-q-F | GCTAATCTTCACTCACCTGAACAG |
| pri-miR263-q-R | GCCTTCATATTACGTATTTGCATTG |
| TTLL4-q-F | GGCCTCAACGAGCCTTACTCAAG |
| TTLL4-q-R | CTGGTTGAGTTGCTGGCAGCC |
| RNA1-q-F | ACACAAAGUCCAGAGGAAAACAA |
| RNA1-q-R | UCACGUUUUAUCCCUUAUGACUU |
| RNA2-q-F | CCCACAGGCACACACACACACTGGCTA |
| RNA2-q-R | ACCCGGATGTGGTGCGTAGCACCATTTC |
| RdRp-q-F | CTTGAGAATGCCAGTGGAAGA |
| RdRp-q-R | CATGCTGGATATTATGCTACCT |
| NP-q-F | GATGAAGTACACAACTGGTC |
| NP-q-R | AGTGCTGATCGTATTGACAGA |
| NP-RNAi-q-F | GATGAAGTACACAACTGGTC |
| NP-RNAi-q-R | AGTGCTGATCGTATTGACAGA |
| RNA1-3’-q-F | ATAACGAGAGGTGGTGTCGTCATCC |
| RNA1-3’-q-R | CCCTTGCATGTTCATGACACATAGTC |
| RNA2-3’-q-F | AAGAATGGGTGTCAGGGAAAGG |
| RNA2-3’-q-R | ACTTTGAGACATGCTACACAAAGTCTG |
| EF2-q-F | GTCTCCACGGATGGGCTTT |
| EF2-q-R | ATCTTGAATTTCTCGGCATACATTT |
| Primers used for expression plasmid construction | |
| NP-F | ATGGGTACCAACAAGCC |
| NP-R | CTAGTCATCTGCACCTTC |
| NP-NotI-F | GCGGCCGCATGGGTACCAAC |
| NP-XbaI-R | TCTAGACTAGTCATCTGCACCTTC |
| RdRp-F | ATGACGACACCACCTCTCG |
| RdRp-R | TCAGAAATCGAACTTATGGTC |
| RdRp-BamHI-F | GGATCCATGACGACACCACCTCTC |
| RdRp-NotI-R | GCGGCCGCTCAGAAATCGAACTTAT |
| P1-TUTR-F | AAGAATTGAAGGATGGCTTCCAAGGTG |
| P1-TUTR-R | AATAACCCGGCGGCCCAAAATGCC |
| P2-TUTR-F | ACCCAGACTTTGTGTAATAACCCGG |
| P2-TUTR-R | ATGGCTTCCAAGGTGTACGACCC |
| *Ls*Pol I-F | GTGTATGATGGCGGCTTCCC |
| *Ls*Pol I-R | GTTGGTTAGGACTGGGACTG |
| *Ls*P1-UTR-F | CACATAGTCAGAGGAAGAATAATT |
| *Ls*P1-UTR-R | GGCCGGCATGGTCCCAC |
| *Ls*P2-UTR-F | ACACAAAGTCTGGGTATAACTTCTTC |
| *Ls*P2-UTR-R | GGCCGGCATGGTCCCAC |
| pLS-R1-EUTR-F | AGATCTTATAACGAGAGGTGGTGTCG |
| pLS-R1-EUTR-R | CCATGGCCCTTGCATGTTCATGACA |
| pLS-R2-EUTR-F | AGATCTTTCACACTAGCCCTGTTG |
| pLS-R2-EUTR-R | CCATGGACTTTGAGACATGCTACAC |
| pLS-R1-UTR-F | CCATGGCTTCCAAGGTGTACG |
| pLS-R1-UTR-R | ACACATAGTCAGAGGAAGAATAATTTTG |
| pLS-R2-UTR-F | CCATGGCTTCCAAGGTGTACG |
| pLS-R2-UTR-R | ACACAAAGTCTGGGTATAACTTC |
| Primers used for RNAi | |
| dsNP-F | AACAAGCCAGCCACTCTA |
| dsNP-R | TCCACAGCCATCTTAACAC |
| dsGFP-F | CACAAGTTCAGCGTGTCCG |
| dsGFP-R | GTTCACCTTGATGCCGTTC |
| Primers used for pri-miR-263a determination | |
| pri-miR263-F | CTCACTCATATTCTCTCTTTCTC |
| pri-miR263-R | CATGCGGTGTGATAACTGTTC |
| Pri-263-KpnI-F | GGGGTACCCTCACTCATATTCTCTCTTTC |
| Pri-263-XhoI-R | CTCGAGCATGCGGTGTGATAACTGTTC |
| Primers used for probe synthesis in FISH | |
| SP6-R1-F | GAATTGATTTAGGTGACACTATAGATAACGAGAGGTGGTGTCG |
| T7-R1-R | GAATTGTAATACGACTCACTATAGGCCCTTGCATGTTCATGAC |
| SP6-PsbP-F | GAATTGATTTAGGTGACACTATAGGGGAAGCCCAAGACGAACAC |
| SP6-PsbP-R | GAATTGTAATACGACTCACTATAGGCTCGGTGATGGTCTTCTTGG |

F, forward primers; R, reverse primers.
