# Supplementary material for: Coordination between terminal variation of the viral genome and insect microRNAs regulates rice stripe virus replication in insect vectors
Source: PLoS Pathog. 2021 Mar 10;17(3):e1009424. doi: 10.1371/journal.ppat.1009424 (PMC7984632; doi:10.1371/journal.ppat.1009424)
Supplement: Attachment — Submitted filename: Responses to comments-R.docx [file ppat.1009424.s007.docx]

The comments are in blue and our responses are in black.

Comments to the Author:

Part I - Summary
Response: Thank the reviewers for their positive comments to our work.

Part II – Major Issues: Key Experiments Required for Acceptance
Reviewer #1:

Line 121 – need to provide rationale/explanation here for why using Human cells to study plant/insect virus
Response: We modify the sentence to provide the explanation for using Human cells: To verify this hypothesis, we used the pPol I plasmid to generate a reverse genetic system for RSV in human embryonic kidney 293T cells due to lack of planthopper cell lines.

Lines 156-169 – the details of acquiring the planthopper Pol I sequence is distracting and is not needed here – suggest moving it to M&M section
Response: We move the details of acquiring the planthopper Pol I sequence to Materials and Methods section as the reviewer suggested. This part of result is simplified as “Considering the species specificity of RNA Pol I promoters, we cloned the Pol I promoter of small brown planthopper to replace the original human Pol I promoter by querying the *L. striatellus* genome [17] based on the high conservation of Pol I promoter sequences [18, 19]. The nucleotides from 15,877 to 15,906 in contig36944 have high identity with the sequences adjacent to the known Pol I transcription initiation sites of other eukaryotes, and T at position -1 and A or G at positions +1 and -2, respectively, are conserved (Fig 2C) [19]. Thus, the 370 bp sequence upstream of the planthopper Pol I transcription initiation site (from -1 to -370) (GenBank registration number MW353861) was cloned as a putative Pol I promoter.”

Line 223 and Fig.3E – in the FISH experiment, how would the miR-263a probe (which is complementary to miR-263a) hybridize with mirR-263a when it is base-paired to the 3’ extension?
Response: FISH is a widely used technique for detecting and locating a specific DNA or RNA sequence in a single cell. The biotin-labeled miR-263a probe and DIG-labeled RNA1 3’-EUTR probe hybridize respectively the single-stranded miR-263a and 3’-EUTR, i.e., free miR-263a and 3’-EUTR. The colocalization of the two types of fluorescence reflects the vicinity of miR-263a and 3’-EUTR of RNA1, which is the basis for their base-pairing.

The Discussion needs improvement:

Provide an integrate findings into working model – cycling from plant to insect.

Response: This is a good suggestion. We summarize a working model cycling from plant to insect in the first paragraph of the Discussion: When planthoppers acquire virions from rice, most virions have normal genomes with complementary 3’- and 5’-UTRs. With viral replication, a 16 nt and a 15 nt sequence are added at the 3’-UTRs of RNA1 and RNA2. The 16 and 15 nt extensions in the 3’ termini of the RSV genomes impair viral promoter activity resulting in the inhibition of viral replication. This inhibitory effect of the 3’ extension of viral RNA1 was decreased by endogenous insect miR-263a, which targets the extended sequence. However, the expression of miR-263a was negatively regulated by RSV infection. This interaction between RSV and insect miRNAs secures the controlled replication of RSV in planthopper reflecting a distinct strategy of adaptation of phytoviruses to the insect vectors.

Gamarnik lab work on Dengue’s 3’UTR in human vs mosquito cells should be discussed.

Response: This work is discussed in the revised manuscript as “The duplicated stem loop structure of the 3’-UTR was beneficial for dengue virus replication in mammalian cells, while deleterious in mosquito cells, suggesting that viral 3’-UTR is involved in insect-host adaptation cycle [25]” in Lines 336-338.

Villordo SM, Filomatori CV, Sanchez-Vargas I, Blair CD, Gamarnik AV. Dengue virus RNA structure specialization facilitates host adaptation. PLoS Pathog. 2015;11: e1004604. PubMed PMID: 25635835.

Do the extensions also inhibit replication in plant cells?

Response: We found in our previous study that the extension sequences were deleterious for viral replication in the plants [10]. We mentioned this information in the Introduction, Line 85.

Possible mechanisms for how the 3’-extension is added should be mentioned.

Response: We discuss the possible mechanisms for how the 3’-extension is added in Lines 341-345: It is possible that the extension sequences are generated by viral RdRp with the template of viral genomic RNAs because the 16 nt and 15 nt extension sequences at the 3’-UTRs of RNA1 and RNA2 are respectively identical to the 8874 nt to 8889 nt fragment in the upstream region of RNA1 and the 3241 nt to 3255 nt fragment in the upstream region of RNA2 [10].

Ideas on how the 3’UTR extension are preferentially added or maintained in insect cells.

Response: We make a discussion on this phenomenon in Lines 338-341: The enrichment of 3’-terminally extended RSV in planthoppers is good for maintaining a limited replication level for virus to strike a balance with insect immune systems without causing severe diseases.

Reviewer #2: Major:
1. Authors should provide detailed information of RSV population before and after given planthopper. The information includes sequences 3’ – terminal of RNA 1 & 2 and percentage of major groups such, no extension, with extensions.

Response: We supplement the detailed information about the relative ratios of 3’-terminally extended types of genomic RNA1 and RNA2 in planthopper and host plant in the Introduction, Lines 81-86: We have demonstrated that the relative ratios of 3’-terminally extended types of genomic RNA1 and RNA2 increased from around 7% to 35% or 49% with viral replication in planthopper and decreased to less than 3% in the late-stage of infection in the plant hosts, and the extension sequences were deleterious for viral replication in the plants [10].

The sequences of 3’-terminal of RNA 1 & 2 are presented in Figure S2.

2. In all of the results presented, RNA or 3’-EUTR level was only detected by Q-RT-PCR. But did not provide how the results were calculated. The authors should use other methods such sequencing or northern blots to confirm their results.

Response: Thanks for this suggestion. We supplement the information about how the results were calculated in Materials and Methods, lines 421 to 427: Viral RNA1 and RNA2 with the 3’-EUTR were identified by amplifying the 3’-UTR containing the extended terminal sequences. The fragments from the inner regions of RNA1 and RNA2 were amplified to measure the total RNA levels of RNA1 and RNA2, respectively. The ratios of RNA1 or RNA2 to the 3’ EUTRs were determined by associating the relative RNA levels of the 3’ EUTRs to those of the RNA levels of RNA1 and RNA2. Primers are listed in S1 Table.

In our previous work, we have used sanger sequencing to verify the 3’ EUTRs [10]. In Figure 3E, FISH experiment showed the 3’-EUTR of RNA1 in the gut cells of viruliferous planthopper.

Part III – Minor Issues
Reviewer #1

Abstract – line29 change to “…due to structural interference with the panhandle structure formed…”
Response: This sentence is rewritten as the reviewer suggested.

Line 73 – change to “.., negative-sense RNA1 and ambisense RNA2, of
Response: This sentence is rewritten as the reviewer suggested.

Line 73 – change semicolon to a period
Response: Corrected.

Figure1C – red bar for NP should have white gap where the scale changes in the y-axis
Response: Thanks for this reminding. Figure 1C is corrected.

Line 119 – change “damage” to “modify”
Response: We replace "damage" with "modify" as the reviewer suggested.

Line 122 – change to “the human RNA polymerase I (Pol I) normally directs the synthesis of ribosomal RNAs (rRNA) [16]. – delete “that function as an RNA template”.
Response: The sentence is corrected to “The pPol I plasmid is driven by the promoter of human RNA polymerase I (Pol I), which normally directs the synthesis of ribosomal RNAs (rRNAs) [16].”

Line 131 – change “, rRNA of…” to “, the Rluc reporter RNA……..was transcribed by Pol I.”
Response: Corrected.

Line 132 – change “Rluc rRNA served…” to “Rluc reporter RNA served..”
Response: Corrected.

Line 151 – change to “inhibit the promoter activity of viral 3’-UTRs in human cells.”
Response: Corrected.

Line 155 – change to “activity in human cells”
Response: Corrected.

Line 195 – please indicate the criteria used to select candidate miRNAs
Response: We supplement the criteria used to select candidate miRNAs in Line 189-192: Two out of 213 putative miRNAs from small brown planthopper, dme-miR-263a-5p (miR-263a) and ame-miR-190 (miR-190), were predicted by both the miRanda and RNAhybrid algorithm to specifically target the 3’-EUTRs of RNA1 and RNA2, respectively.

Fig3 – please comment on the endogenous levels of miR-263a vs the concentrations being injected
Response: We add the description of injection of miRNA agomirs in the Result, Lines 207-211: To verify the interaction between miR-263a and the 3’-EUTR of viral RNA1 *in vivo*, we performed RNA immunoprecipitation (RIP) assay using an in-house generated planthopper anti-argonaute 1 (Ago1) monoclonal antibody [21] in viruliferous planthoppers after injected with agomirs of miR-263a or miR-190, which made the endogenous levels of the two miRNAs increase by 4- or 1.3-fold (Fig 3C).

For Figs3, 4 and 5 – might want to consider standardizing the color scheme of the graphs – Figs 3 and 5 are in black & white, whereas Fig 4 is in color.
Response: Thanks for this suggestion. Figure 4 is changed to black and white.

Line 353 – please expand more specifically on how the miR interaction and Ago binding could “relieve” the inhibitory effect of the 3’-extension

Response: We rewrite the sentence to give a hypothesis: The miR-263a, possibly with the aid of Ago1, covers the extension sequences, rendering the UTRs-formed panhandle structure exposed to viral RdRp for binding.

Reviewer #2: 
1. In Fig. 1 A to D, the sampling time should be provided either in results or fig legends.

Response: We supplement the sampling time in the figure legend of Fig. 1: Viruliferous fourth-instar nymphs and rice leaves with obvious stripe disease symptoms were sampled for virus extraction.

2. In Fig. 2 B, how much of P1-WT and P2-WT were added in these two experiments?

Response: The detailed information about the amount of various recombinant plasmids transfected to cells can be found in Materials and Methods, Line 452. We specify the pPol I plasmids with “including P1/P2-WT, P1/P2-EUTR, and P1/P2-TUTR”.

3. Line 35, rice strip virus is member of Tenuivirus genus, not Phytoreovirus.

Response: Here, we use “phytoviruses”, not “Phytoreoviruses”.

4. Line 41 “rice strip viruses” should be corrected to “rice strip virus”.

Response: Corrected.

5. Line 77 “an RdRp” should be “a RdRp”.

Response: “an” is usually used in front of a word beginning with a vowel pronunciation, like “R”. We read “RdRp” in a way of letter by letter.

Reviewer #3:

Line 2 and the rest of the manuscript: I believe “insect vectors” is more commonly used than “vector insects”. The latter doesn’t read as well as the former. In a small number of places in the manuscript “insect vectors” has been used. It would be good to have consistency, either way.

Response: Thanks for this suggestion. “vector insects” has been changed to “insect vectors” throughout the manuscript.

Line 27: insert the scientific name of the planthopper as it is not mentioned in the title or the abstract.

Response: “*Laodelphax striatellus*” is added in the abstract.

Line 56: one of the major…

Response: Corrected.

Line 100 and 109: technically, the amount of RNA is not equivalent to the number of virions. Virion should be replaced with viral RNAs

Response: Corrected.

Line 139: with the assistance of….

Response: Corrected.

Line 230: the authors should be consistent with the usage of mimic/agomir. There are other instances of interchangeable usage of the terms.

Response: miRNA agomir is different from mimic. Agomir is chemically-modified miRNA mimic with modified antisense strand: 2 phosphorothioates at the 5′ end, 4 phosphorothioates at the 3′ end, 3′ end cholesterol group, and full-length nucleotide 2′-methoxy modification. Therefore, agomir has a higher stability than mimic and is more suitable for application in experiments *in vivo*. We used miRNA mimic for experiments in 293T cells, and miRNA agomir for experiment in planthoppers.

Line 285: injected into…

Response: Corrected.

Line 456 and 583: Functional analysis of …..
Response: Corrected.

Fig. 3E: The figure is not really clear in that it doesn’t have a defined nucleus and cytoplasmic region. Does the enlarged image in the lower row represent one cell?

Response: We used Hoechst to stain nuclei (blue). The enlarged image contains more than one cell. Colocalization of miR-263a and the 3’-EUTR of RNA1 in the gut cells was observed in the cytoplasm and partial region of the nucleus.
